# Association of serum 25(OH)D with Cathepsin K levels in gingival crevicular fluid and saliva in periodontal health and disease: a cross-sectional study

**DOI:** 10.1186/s12903-025-07637-0

**Published:** 2026-01-07

**Authors:** Ali Batuhan Bayırlı, Mehmetcan Uytun, Ercan Saruhan, İsmail Kırlı, Ayla Öztürk

**Affiliations:** 1 Department of Periodontology, School of Dentistry, Mugla Sıtkı Kocman University, Mugla, 48000 Türkiye; 2 Department of Biochemistry, School of Medicine, Mugla Sıtkı Kocman University, Mugla, Türkiye; 3 Department of Internal Medicine, School of Medicine, Mugla Sıtkı Kocman University, Mugla, Türkiye; 4 Department of Periodontology, School of Dentistry, Ankara University, Ankara, 06500 Türkiye

**Keywords:** Periodontitis, Vitamin D, Cathepsin K, Gingival crevicular fluid, Saliva

## Abstract

**Background:**

This study investigates the relationship between serum vitamin D concentration and Cathepsin K (CatK) levels in gingival crevicular fluid (GCF) and saliva in both periodontal health and disease.

**Methods:**

Sixty-nine participants were categorized into four groups based on periodontal status and serum vitamin D levels: vitamin D deficient with periodontal health (*n* = 18), vitamin D sufficient with periodontal health (*n* = 18), vitamin D deficient with periodontitis (*n* = 17), and vitamin D sufficient with periodontitis (*n* = 16). Clinical periodontal parameters were recorded. CatK levels in GCF and saliva were measured using enzyme-linked immunosorbent assays.

**Results:**

GCF and salivary CatK levels were higher in the vitamin D deficient group in both health and periodontitis, with the highest levels in the vitamin D deficient periodontitis group and the lowest in the vitamin D sufficient periodontal health group (*p* < 0.001). A significant negative correlation between serum vitamin D levels and both GCF and salivary CatK levels, while CatK levels positively correlated with all clinical periodontal parameters (*p* < 0.001). Serum vitamin D, probing pocket depth (PPD), and bleeding on probing explained 83.6% of the variation in GCF CatK levels (R² = 0.836, *p* < 0.001), while serum vitamin D, PPD, and gingival index accounted for 83.4% of the variation in salivary CatK levels (R² = 0.834, *p* < 0.001).

**Conclusion:**

Within the limitations of this study, vitamin D deficiency appeared to be associated with higher Cathepsin K levels in gingival crevicular fluid and saliva, particularly among individuals with periodontitis. The observed negative correlation between serum vitamin D and CatK, together with the positive associations of CatK with clinical periodontal parameters, suggests a possible link between systemic vitamin D status, local biomarker activity, and periodontal disease severity. These findings may indicate a potential role of vitamin D in periodontal host modulation, which warrants further investigation in larger, longitudinal studies.

**Supplementary Information:**

The online version contains supplementary material available at 10.1186/s12903-025-07637-0.

## Background

Oral health is key in overall health. The periodontium, which support the teeth, consists of gingiva, periodontal ligament, cementum and alveolar bone, functioning as a unit [1]. According to the World Health Organization, health encompasses complete physical, mental and social well-being, and in this context, periodontal health refers to the absence of inflammatory periodontal disease [2, 3]. Periodontitis is a chronic inflammatory disease that is associated with dysbiotic plaque biofilms and is characterized by the progressive destruction of the supporting tissues of the teeth. The main clinical findings include clinical attachment loss, alveolar bone destruction, periodontal pocket formation, and gingival bleeding [4]. Due to its high prevalence, periodontitis is a significant public health concern, leading to tooth loss, reduced quality of life, and a negative impact on overall health. Although microbial dental plaque is the primary cause of the initiation and progression of this process, various hormones and enzymes play a role in the transition from gingivitis to periodontitis [5–7]. Among these, vitamin D and Cathepsin K (CatK) play crucial roles [8, 9].

Vitamin D is a fat-soluble secosteroid that exists in two forms, D2 and D3. Human skin synthesizes vitamin D3 upon exposure to UVB radiation from sunlight, which is then converted into its active form, 1,25-dihydroxyvitamin D3 (1,25(OH)2D3) [10]. Beyond its role in calcium homeostasis, 1,25(OH)2D3 plays an important role in immune response and regulation of inflammation by influencing cellular proliferation and differentiation through the vitamin D nuclear receptor (VDR) [10, 11]. Vitamin D status is assessed by serum 25-hydroxyvitamin D3 (25(OH)D3) levels, with a normal range of 30–50 ng/mL [11]. Several studies have linked vitamin D deficiency with periodontal disease, particularly with increased severity of chronic inflammation [12–16].

Vitamin D plays multifaceted roles in regulating the immune system, exerting strong anti-inflammatory effects [17]. Deficiency increases the frequency and severity of chronic inflammatory diseases such as periodontitis, cardiovascular diseases, inflammatory bowel diseases and autoimmune disorders [13, 16]. Vitamin D supplementation has been shown to reduce the severity of these diseases and lower pro-inflammatory mediator levels in deficient patients [13, 18].

Another important biomarker associated with periodontal disease is CatK. Cathepsins are enzymes belonging to the lysosomal cysteine protease family. CatK is secreted by active osteoclasts especially during bone resorption. It breaks down collagen and other matrix proteins [19]. CatK plays a critical role in bone resorption; however, it can prevent bone resorption without affecting bone formation, making it a key target for the development of antiresorptive drugs [20]. Like vitamin D, CatK influences bone metabolism [21–23]. Additionally, vitamin D has been shown to stimulate CatK expression along with other agents such as tumor necrosis factor (TNF) and parathyroid hormone, which promote osteoclast formation and differentiation [24].

Since periodontitis is characterized by alveolar bone loss, the relationship between CatK and periodontitis has garnered considerable interest among researchers. CatK can be detected in saliva, gingival crevicular fluid (GCF) and gingival tissue. It has been reported that CatK levels in GCF are higher in periodontitis patients than in periodontally healthy individuals [25–28].

Host biomarkers play a crucial role in oral ecology through receptors for microbiome metabolites, as well as through inflammatory mediators and enzymes. Biomarker analysis not only provides insights into pathogenic processes but also forms the basis for precision medicine, allowing disease activity to be monitored with reliable markers [29, 30]. Periodontitis is known to progress through three stages: inflammation, connective tissue destruction and bone resorption, and the detection of specific biomarkers at each stage serves as a critical guide in the diagnosis and management of the disease. In cases where traditional diagnostic methods are insufficient, meticulous monitoring of biomarkers contributes to individualized treatment approaches by providing a more accurate assessment of disease activity [30, 31]. Therefore, biomarkers in GCF and saliva show promise for more objective and reliable assessment of periodontal status [32–34]. While GCF has been used to detect inflammation indicators, saliva has the advantage of being easily and non-invasively collected [34–37]. Furthermore, many biomarkers identified in GCF can also be found in saliva, making saliva an important diagnostic tool [34].

The roles of both vitamin D and CatK in the inflammatory process of periodontal diseases, as well as studies examining the levels, effects, and roles of these biomarkers separatelly at variying stages of periodontal disease, guided our study. However, no studies have yet evaluated the effects of these biomarkers together. While many studies have explored the role of CatK in the pathogenesis of periodontal diseases, this study, which evaluates vitamin D deficiency and CatK levels together, aims to fill an important gap in the field. We hypothesized that serum vitamin D deficiency is associated with elevated CatK levels in GCF and saliva, and that these alterations correlate with clinical periodontal parameters. The aim of the study was to examine the relationship between serum vitamin D concentration and CatK levels in GCF and saliva in periodontal health and disease.

## Methods

### Study design and participants

This study was approved by the Izmir Bakırçay University Non-Interventional Clinical Research Ethics Committee on 09.10.2024 with decision number 1798 and was conducted in accordance with the principles of the Declaration of Helsinki. The individuals included in the study were informed in detail about the purpose and content of the study. Written informed consent was obtained from the participants. The study was conducted and reported in accordance with the STROBE guidelines. Clinical and radiographic examinations were performed at the Department of Periodontology, Faculty of Dentistry, Muğla Sıtkı Koçman University. A total of 69 participants who met the following criteria were included in the study: (i) 25–40 years of age; (ii) systemically healthy individuals; (iii) no regular use of systemic or local medications; (iv) no periodontal treatment in the last six months; (v) non-smokers. These criteria were applied to minimize potential confounding factors, as systemic diseases, medications, and smoking are known to influence both vitamin D metabolism and CatK expression. Thus, a more homogeneous population was ensured, allowing the observed associations to reflect primarily the effects of periodontal status and vitamin D levels.

### Study groups

Individuals were grouped by periodontal status and vitamin D levels into four groups: vitamin D deficient with periodontal health, vitamin D sufficient with periodontal health, vitamin D deficient with periodontitis, and vitamin D sufficient with periodontitis. The diagnosis of periodontal status of participants in the study groups was categorized in accordance with the 2017 European Federation of Periodontology and American Academy of Periodontology classification [3, 4]. During October 2024, the volunteers had their serum 25(OH)D levels measured at Muğla Sıtkı Koçman University Faculty of Medicine, Department of Internal Medicine and were then referred to the Periodontal Department. 25(OH)D levels above 30 ng/mL were considered sufficient, and levels below 20 ng/mL were regarded as vitamin D deficiency [38, 39]. Both GCF and saliva samples were collected from each participant.

#### Vitamin D deficient periodontal health group (*n* = 18 GCF samples, *n* = 18 saliva samples)

This group consisted of 18 periodontally healthy individuals. Inclusion criteria were: no clinical attachment loss in all teeth, pocket depth ≤ 3 mm in all teeth, no radiographic bone loss and less than 10% bleeding during probing, and 25(OH)D level below 20 ng/mL.

#### Vitamin D sufficient periodontal health group (*n* = 18 GCF samples, *n* = 18 saliva samples)

This group consisted of 18 periodontally healthy individuals. The inclusion criteria were: no clinical attachment loss in all teeth, pocket depth ≤ 3 mm in all teeth, no radiographic interproximal bone loss and less than 10% bleeding during probing, and 25(OH)D level above 30 ng/mL.

#### Vitamin D deficient periodontitis group (*n* = 17 GCF samples, *n* = 17 saliva samples)

This group consisted of 17 patients with stage III, grade B periodontitis. Inclusion criteria were: loss of up to 4 teeth due to periodontal disease, clinical attachment loss of at least 5 mm in any tooth, least 6 mm pocket depth in any tooth, radiographic bone loss extending to the middle or apical third of the root, and 25(OH)D level below 20 ng/mL. Grade B was determined according to the bone loss/age ratio, in the absence of heavy smoking or uncontrolled diabetes.

#### Vitamin D sufficient periodontitis group (*n* = 16 GCF samples, *n* = 16 saliva samples)

This group consisted of 16 stage III, grade B periodontitis patients. The inclusion criteria were: loss of up to 4 teeth due to periodontal disease, clinical attachment loss of at least 5 mm in any tooth, least 6 mm pocket depth in any tooth, radiographic bone loss extending to the middle or apical third of the root, and 25(OH)D levels above 30 ng/mL. Grade B was determined according to the bone loss/age ratio, in the absence of heavy smoking or uncontrolled diabetes.

### Periodontal examination

All participants underwent clinical and radiographic examinations. Periodontal examination included evaluation of plaque index (PI), gingival index (GI), bleeding on probing (BOP), probing pocket depth (PPD) and clinical attachment level (CAL). Measurements were made at six points on each tooth. A Williams periodontal probe (Hu-Friedy Manufacturing Co. Inc., Chicago, IL, USA) was used for these measurements. These measurements were performed by two periodontologists (A.B.B. and M.U.), with 10 and 5 years of clinical experience in periodontology, respectively. Prior to the study, calibration was completed on 20 patients with stage III periodontitis. Intra- and inter-examiner reliability was assessed using the intra-class correlation coefficient (ICC). Intra-examiner ICC values were 0.91 (PPD) and 0.90 (CAL) for A.B.B. and 0.91 (PPD) and 0.93 (CAL) for M.U, Inter-examiner ICC values were calculated as 0.90 (PPD) and 0.89 (CAL). As a result, the measurements were considered reliable. Both examiners were blinded to the laboratory results during the clinical examinations. In addition, all biochemical analyses were performed on coded samples, and the biochemist conducting the ELISA assays was blinded to the clinical status and group allocation of the participants.

### Saliva sample acquisition

Saliva samples were obtained from all participants in each study group. Saliva samples for biochemical analysis of CatK levels were collected non-invasively by passive saliva technique [40]. Unstimulated saliva samples were collected in Eppendorf tubes in the morning to catch the standard. Before providing 2–4 mL saliva samples, participants were asked to rinse their mouths with water only for two minutes to remove food debris. After the collected saliva samples were centrifuged at 1000×g for 10 min, the supernatants were transferred to Eppendorf tubes and stored at -80 °C in the deep freezer in the Biochemistry Research Laboratory of Muğla Sıtkı Koçman University Faculty of Medicine until biochemical analysis.

### GCF sample acquisition

GCF samples were obtained from all participants in each study group. In the periodontal health group, GCF samples were collected from the vestibular surfaces of maxillary central incisors. Participants in the control group had no missing teeth. In the periodontitis group, sample sites were selected from teeth with the deepest periodontal pockets (PPD ≥ 6 mm). For each patient, one site from one tooth was used for sample collection. Before GCF sample collection, supragingival plaque was removed with a sterile scaler (Hu-Friedy Manufacturing Co. Inc., Chicago, IL, USA) without touching the gingival margin. The tooth was isolated with a roll of cotton wool and a saliva absorbent, and then dried with a sterile 2 × 2 gauze. A paper strip was used for each site. 2 × 14 mm paper strips (Periopaper^®^, Pro Flow Inc., Amityville, NY, USA) of standardized size and absorbency were used for GCF collection. These special paper strips were placed in the gingival sulcus and left for 30 s. Paper strips contaminated with saliva or blood were excluded from the evaluation. The paper strips were placed in Eppendorf tubes containing 300 µL phosphate buffered saline. Samples were centrifuged at 1000×g for 10 min. The supernatants obtained from the GCF were separated for study. The samples were mixed thoroughly and stored at -80 °C until the day of the study.

### Cathepsin K measurements

GCF and saliva CatK concentrations were measured by using human CatK enzyme-linked immunosorbent assays (ELISA) kit (Cat# E0833Hu, BT-Laboratory, Shanghai, China) according to the instructions of the manufacturer. The laboratory personnel performing the ELISA analyses were blinded to the clinical periodontal status and group allocation of the participants. Measurements were carried out using an ELISA plate reader (Multiskan GO microplate reader, Thermo Fisher Scientific, Waltham, MA, USA). CatK assay sensitivity was 0.18 pmol/L with inter-assay and intra-assay coefficients of variation less than 10%. In accordance with the manufacturer’s protocol, CatK measurements were performed as single readings rather than duplicate or triplicate analyses.

All assays were performed according to the manufacturer’s instructions. Standard curves were generated for each plate, and all sample absorbance values fell within the range of the standard curve. Standard curves for each ELISA plate showed excellent linearity (R² > 0.99). Representative absorbance values and corresponding concentrations are provided in Supplementary Figure S1 and S2.

### Statistical analysis

IBM SPSS Statistics Version 21 package program was used in the analysis. Statistical methods were used to determine the differences between the study groups. Data normality was evaluated using the Shapiro–Wilk test, which confirmed that the assumption of normal distribution was satisfied across all groups (*p* > 0.05). The assumption of homogeneity of variances was assessed using Levene’s test, which indicated significant differences in group variances (*p* < 0.05). Given this violation of the homogeneity assumption, the Welch ANOVA—robust to unequal variances—was employed for subsequent analysis. Post-hoc pairwise comparisons between groups were conducted using Welch’s t-test with Bonferroni correction for multiple comparisons. In addition, the relationship between the measurements was determined by Spearman correlation coefficient and chi-square test. Finally, stepwise regression analysis was performed. A value of *p* < 0.05 was used for statistical significance.

#### Power analysis

To calculate the sample size, the data from Mogi et al. [41] were used and power analyses were conducted using the G Power statistical program. Power analysis indicated that a total of 64 individuals (16 per group) were required to achieve a minimum effect size level of 1.04 f with a 95% CI and 80% power.

## Results

In our study, GCF and saliva samples from 69 patients were analyzed. 49.3% of the participants were female and 50.7% were male. The age of the patients ranged from 25 to 38 years. The mean age was calculated to be 31.95 ± 3.40 years. The gender distribution between the groups did not show a statistically significant difference (*p* = 0.961). Similarly, the age distribution showed no significant difference between the groups (*p* = 0.588).

Table 1 shows the distribution of age, gender, clinical periodontal parameters and biochemical parameters according to the groups. Significant differences (*p* < 0.001) were found in periodontal parameters, with the highest values in periodontitis groups and the lowest values in periodontally healthy groups. When GCF and salivary CatK levels were analyzed, the highest values were found in the vitamin D deficient periodontitis group and the lowest values were found in the vitamin D sufficient periodontal health group (*p* < 0.001). When serum vitamin D levels were compared within the periodontitis and periodontal health groups, statistically significant differences were found between the vitamin D sufficient and deficient groups (*p* < 0.001).

**Table 1:** Clinical, biochemical and demographic data

		Periodontal Health		Periodontitis		
Parameters		**25(OH)D < 20** **(*n*** **= 18)**	**25(OH)D ≥ 30** **(*n*** **= 18)**	**25(OH)D < 20** **(*n*** **= 17)**	**25(OH)D ≥ 30** **(*n*** **= 16)**	***p*** **-value**
**Gender**						
** Female (Frequency)**		9 (%50)	9 (%50)	9 (%53)	7 (%44)	0.961
** Male (Frequency)**		9 (%50)	9 (%50)	8 (%47)	9 (%56)	
**Age**		31.11 ± 3.54	31.88 ± 3.83	32.23 ± 2.68	32.68 ± 3.51	0.588
**PI**		0.82 ± 0.19^a^	1.06 ± 0.22^a^	1.94 ± 0.59^b^	2.24 ± 0.23^b^	**< 0.001**
**GI**		0.83 ± 0.21^a^	1.11 ± 0.26^a^	1.67 ± 0.33^b^	2 ± 0.31^c^	**< 0.001**
**BOP (%)**		8.35 ± 1.53^a^	8.68 ± 1.11^a^	45.82 ± 18.9^b^	59.16 ± 12.59^c^	**< 0.001**
**PPD (mm)**		1.87 ± 0.32^a^	1.98 ± 0.36^a^	3.77 ± 0.59^b^	3.84 ± 0.32^b^	**< 0.001**
**CAL (mm)**		0.63 ± 0.14^a^	0.78 ± 0.14^a^	2.01 ± 1.19^b^	3.6 ± 0.92^c^	**< 0.001**
**GCF CatK (pmol/L)**		9.93 ± 1.92^a^	3.66 ± 1.40 ^b^	30.60 ± 4.45 ^c^	21.60 ± 2.44 ^d^	**< 0.001**
**Saliva CatK (pmol/L)**		9.98 ± 1.59^a^	4.15 ± 1.79^b^	29.78 ± 2.95^c^	16.88 ± 4.20^d^	**< 0.001**
**Serum Vitamin D (ng/mL)**		9.36 ± 4.27^a^	47.92 ± 8.02^b^	6.46 ± 2.45^a^	50.11 ± 8.22^b^	**< 0.001**

Values are presented as mean ± standard deviation (SD). Different superscript letters (a, b, c, d) indicate statistically significant differences between groups at *p* < 0.05 (Welch ANOVA with Welch’s t-test and Bonferroni correction for multiple comparisons). Bold values indicate statistical significance (*p* < 0.05)

*Abbreviations*: *GCF* gingival crevicular fluid, *PI* plaque index, *CatK* cathepsin K, *GI* gingival index, *BOP* bleeding on probing, *PPD* probing pocket depth, *CAL* clinical attachment level

Table 2 shows the results of Spearman’s rho correlation analysis of biochemical and clinical periodontal parameters. Spearman’s rho correlation coefficient was used to evaluate the relationship between clinical parameters and serum vitamin D levels and GCF and salivary CatK levels. A statistically significant negative correlation was observed between GCF and saliva CatK levels and serum vitamin D levels (*p* < 0.001). However, a statistically significant positive correlation was observed between GCF and saliva CatK levels and all clinical periodontal parameters (*p* < 0.001). A positive correlation was observed only between serum vitamin D level and GI among clinical periodontal parameters (*p* < 0.05). Nevertheless, in stepwise regression analyis this association disappeared after adjusting for PI, CAL and PPD.

**Table 2:** Spearman correlation analysis between biochemical parameters and clinical parameters

Parameters (*n* = 69)		GCF Cat K	Saliva Cat K	Vitamin D
**Age**	r	0.049	0.059	0.153
**PI**	r	**0.595**	**0.561**	0.215
**GI**	r	**0.573**	**0.539**	**0.244**
**BOP**	r	**0.705**	**0.653**	0.066
**PPD**	r	**0.73**	**0.723**	0.066
**CAL**	r	**0.597**	**0.581**	0.235
**GCF CatK**	r	1	**0.899**	**-0.417**
**Saliva CatK**	r	**0.899**	1	**-0.467**

Bold values indicate statistical significance (*p* < 0.05)

*Abbreviations*: *r* Spearman’s rank correlation, *n* total sample size, *GCF* gingival crevicular fluid, *PI* plaque index, *CatK* cathepsin K, *GI* gingival index, *BOP* bleeding on probing, *PPD* probing pocket depth, *CAL* clinical attachment level

Table 3 presents the results of stepwise regression analysis performed to determine the effects of serum vitamin D level, age, gender and clinical periodontal parameters on CatK levels in GCF and saliva. According to the results of stepwise regression analysis, serum vitamin D level, PPD, BOP and GI had statistically significant effects on CatK levels (*p* < 0.05). The explanatory power of the regression model was found to be high, *R* = 0.914 and R^2^ = 0.836 for CatK level in GCF and *R* = 0.913 and R^2^ = 0.834 for salivary CatK level. These findings indicate that the independent variables explained 83.6% and 83.4% of the variation in GCF and salivary CatK levels, respectively. These results suggest that serum vitamin D level has a negative relationship with CatK. In addition, PPD and BOP are positively associated with GCF CatK levels and PPD and GI parameters are positively associated with salivary CatK levels.

**Table 3:** Stepwise regression analysis of cathepsin K levels in GCF and saliva in relation to clinical and biochemical parameters

Model	Dependent variables	Independent variables	B	SE	Beta	t	*p*	Tolerance	VIF
1	GCF CatK	Constant	1.526	2.146		0.711	0.48		
		Serum Vitamin D	-0.209	0.026	-0.415	-8.169	**< 0.001**	0.977	1.023
		PPD	5.736	0.996	0.542	5.759	**< 0.001**	0.284	3.52
		BOP	0.144	0.041	0.332	3.513	**0.001**	0.281	3.556
			*F = 110.66; p < 0.001*			*R* = 0.914		*R*^*2*^ *= 0.836*	
2	Saliva CatK	Constant	0.286	1.608		0.178	0.859		
		Serum Vitamin D	-0.26	1.608	-0.559	-10.010	**< 0.001**	0.821	1.218
		PPD	6.108	0.848	0.625	7.202	**< 0.001**	0.339	2.95
		GI	3.42	1.703	0.182	2.008	**0.049**	0.312	3.21
			*F = 108.73; p < 0.001*			*R* = 0.913		*R*^*2*^ *= 0.834*	

Bold values indicate statistical significance (*p* < 0.05)

*Abbreviations*: *B*, unstandardized regression coefficient, *Beta* standardized regression coefficient, *SE* standard error, *GCF* gingival crevicular fluid, *CatK* Cathepsin K, *GI* gingival index, *BOP* bleeding on probing, *PPD* probing pocket depth

## Discussion

The goal of this study is to investigate the relationship between serum vitamin D levels and the levels of CatK in GCF and saliva in periodontal tissues. Our main findings indicate that the levels of CatK in both saliva and GCF were higher in the vitamin D deficient group compared to the vitamin D sufficient group, in both the healthy group and the periodontitis group. The highest CatK levels were observed in the vitamin D deficient periodontitis group, followed by the vitamin D sufficient periodontitis group, the vitamin D deficient healthy group, and finally, the vitamin D sufficient healthy group. Additionally, a negative relationship between vitamin D levels and CatK was observed, with CatK levels decreasing as vitamin D concentration increased. Clinical parameters (CAL, PPD, BOP, GI) were positively associated with CatK. Regression analysis explained 83.6% of the total variance of these variables in GCF and 83.4% in saliva.

These results suggest that CatK levels are elevated in parallel with the severity of periodontal disease. Therefore, the potential role of CatK in periodontal tissues may be of importance. The data obtained suggest that CatK may be involved in the progression of periodontal disease through its association with osteoclastic activity and inflammation. Furthermore, vitamin D deficiency may be associated with the progression of periodontitis, possibly through higher CatK release.

CatK is an extracellular matrix degrading enzyme expressed by osteoclasts and plays a role in bone resorption [28, 42]. Two studies, similar to ours, showed that CatK levels in GCF were higher in periodontitis patients than in periodontally healthy individuals [28, 41]. According to the researchers, this suggests that CatK may also be involved in osteoclast-mediated bone resorption. In another study by Gajendran et al., a positive correlation was found between CatK and receptor activator of nuclear factor β ligand (RANKL) in periodontitis patients [43]. This finding is consistent with our results, given the role of RANKL in alveolar bone resorption. Similarly, CatK may be associated with the severity of periodontal disease, based on the positive correlation of GCF and salivary CatK levels with all clinical periodontal parameters in our study. This finding is consistent with previous studies suggesting that CatK plays a role in extracellular matrix degradation and may be a potential indicator of periodontal tissue destruction [26, 27]. Similar results have been obtained in experimental studies investigating the role of CatK in bone resorption. Chen et al. [44] reported that suppressing CatK expression using adeno-associated virus-mediated gene silencing significantly prevented osteoclast-mediated bone resorption in experimental periodontitis studies. This finding suggests that CatK plays a critical role in osteoclast-mediated bone resorption and is an important factor triggering bone loss in diseases such as periodontitis.

Vitamin D plays roles in the pathogenesis of periodontal disease, including the regulation of pro-inflammatory cytokine production, stimulation of antimicrobial peptide secretion, and activation of hydrogen peroxide release in monocytes [13]. The innate immune response involves antimicrobial peptide secretion against periodontal pathogens. Antimicrobial peptides such as β -defensins and cathelicidins play critical roles in neutralizing bacterial endotoxins and lipopolysaccharides [14, 45]. Additionally, vitamin D contributes to the inhibition of proteinase expression that may lead to tissue destruction [46]. The cathepsin family is a subgroup of proteinases [19, 47]. To the best of our knowledge, no study has investigated the relationship between CatK and vitamin D in periodontal tissues. Our study is the first in this regard.

A stepwise regression analysis was used, in which only variables with statistically significant contributions were retained in the final model. The regression models explained approximately 83% of the variance in both GCF and salivary CatK levels, indicating a strong explanatory power. The analysis showed that serum vitamin D levels were negatively associated with GCF CatK, suggesting that vitamin D may be linked to reduced periodontal tissue destruction through its potential role in suppressing osteoclastic activity. In contrast, PPD and BOP were positively associated with CatK levels, suggesting that CatK expression tends to be higher in the presence of greater periodontal inflammation and tissue breakdown. Similarly, serum vitamin D was negatively associated with salivary CatK levels, whereas PPD and GI were positively associated. Among these predictors, PPD emerged as the strongest independent determinant in both GCF and saliva models, followed by serum vitamin D. These findings indicate that deep periodontal pockets and inflammation are associated with higher CatK expression, providing important clues to the possible regulatory role of vitamin D in periodontal disease. Age and gender were also included in the analysis; however, their inclusion did not alter the significance or direction of the observed associations.

To ensure that these associations were assessed with methodological rigor, CatK levels were quantified based on the total protein collected during a standardized 30-second interval. This approach was chosen because Lamster et al. [48] demonstrated that expressing biomarker levels as absolute amounts in a fixed collection period provides a more sensitive and reliable assessment of changes in GCF composition. Their findings further indicated that data expressed as concentrations within a standard GCF volume may mask biologically relevant variations, as the measured concentrations did not always parallel the clinical presentation. Therefore, using a time-standardized collection enhances the validity of biomarker evaluation by minimizing discrepancies between clinical status and GCF composition. A notable strength of this study is the multilayered blinding approach implemented across different stages of the research process. The clinician performing the periodontal examinations was blinded to the participants’ vitamin D status, minimizing expectation bias during clinical measurements. Additionally, both the laboratory analyst and the statistician were blinded to group allocation during biochemical quantification and the initial stages of data analysis, ensuring objective handling of laboratory data and statistical outputs. This multi-step blinding structure enhances the methodological rigor of the study and strengthens the reliability of its findings.

Nevertheless, this study has a few limitations. First, the cross-sectional design of the study prevents the determination of causality between the variables. Longitudinal and intervention studies are needed to assess the long-term impact of vitamin D and CatK on the progression of periodontal disease. Second, the sample size is relatively limited, and studies conducted on larger and more diverse populations could enhance the generalizability of the findings. In addition, CatK measurements were performed as single readings rather than duplicate analyses, although assay reliability was supported by low intra- and inter-assay variation and excellent standard curve linearity. Vitamin D levels were measured from serum, and the lack of direct assessment of local vitamin D levels in periodontal tissues may restrict a full understanding of the biological mechanisms. In addition, potential confounding variables such as genetic factors, dietary habits, and sun exposure, which could influence the development and progression of periodontal disease, were not considered. Future studies investigating protein expression levels in periodontal tissues could contribute to a better understanding of the role of CatK in pathogenesis. Considering all these limitations, it is recommended that future research should explore these relationships in more depth through larger-scale, prospective studies incorporating mechanistic approaches.

## Conclusion

This study evaluated the relationship between serum vitamin D levels and GCF and salivary CatK levels, suggesting that vitamin D deficiency may be associated with alterations in periodontal destruction markers. The findings showed that CatK levels in GCF and saliva were significantly higher in individuals with low serum vitamin D levels. Furthermore, the positive correlation of CatK levels with clinical periodontal parameters suggests that this enzyme may contribute to periodontal disease progression. In conclusion, the relationship between serum vitamin D levels and GCF and salivary CatK levels may be clinically relevant for periodontal disease management. Future studies are needed to further clarify these associations and to explore whether vitamin D supplementation could influence periodontal disease processes and treatment outcomes.

## Supplementary Information

Supplementary Material 1: Supplementary Figure S1. Standard curve of Cathepsin K ELISA. Representative standard curve obtained for Cathepsin K ELISA, demonstrating excellent linearity (R² = 0.997). Fitted regression line and residuals are shown. Signal values correspond to optical density at 450 nm (OD450).

Supplementary Material 2: Supplementary Figure S2. Representative absorbance values and standard curve-derived concentrations for Cathepsin K ELISA (Plate 1). Raw absorbance values at 450 nm, blank-subtracted signals, and corresponding concentrations interpolated from the standard curve are presented for both standard samples and representative unknown GCF samples. These data illustrate the transparency of the quantification process and confirm that all sample values fell within the dynamic range of the assay.

## Data Availability

The datasets used and/or analyzed during the current study are available from the corresponding author on reasonable request.

## Abbreviations

25(OH)D: 25-hydroxyvitamin D
1,25(OH)₂D₃: 1,25-dihydroxyvitamin D₃
AAP: American Academy of Periodontology
ANOVA: Analysis of Variance
BOP: Bleeding on Probing
CAL: Clinical Attachment Level
CatK: Cathepsin K
CI: Confidence Interval
ELISA: Enzyme-Linked Immunosorbent Assay
EFP: European Federation of Periodontology
GCF: Gingival Crevicular Fluid
GI: Gingival Index
ICC: Intraclass Correlation Coefficient
PPD: Probing Pocket Depth
PI: Plaque Index
PBS: Phosphate Buffered Saline
R: Regression coefficient
R²: Coefficient of Determination
RANKL: Receptor Activator of Nuclear Factor Kappa-Β Ligand
SD: Standard Deviation
SE: Standard Error
SPSS: Statistical Package for the Social Sciences
STROBE: Strengthening the Reporting of Observational Studies in Epidemiology
TNF: Tumor Necrosis Factor
VDR: Vitamin D Receptor
VIF: Variance Inflation Factor
