# Supplementary figures and images for: Association of serum 25(OH)D with Cathepsin K levels in gingival crevicular fluid and saliva in periodontal health and disease: a cross-sectional study
Source: BMC Oral Health. 2026 Jan 7;26:237. doi: 10.1186/s12903-025-07637-0 (PMC12870205; doi:10.1186/s12903-025-07637-0)

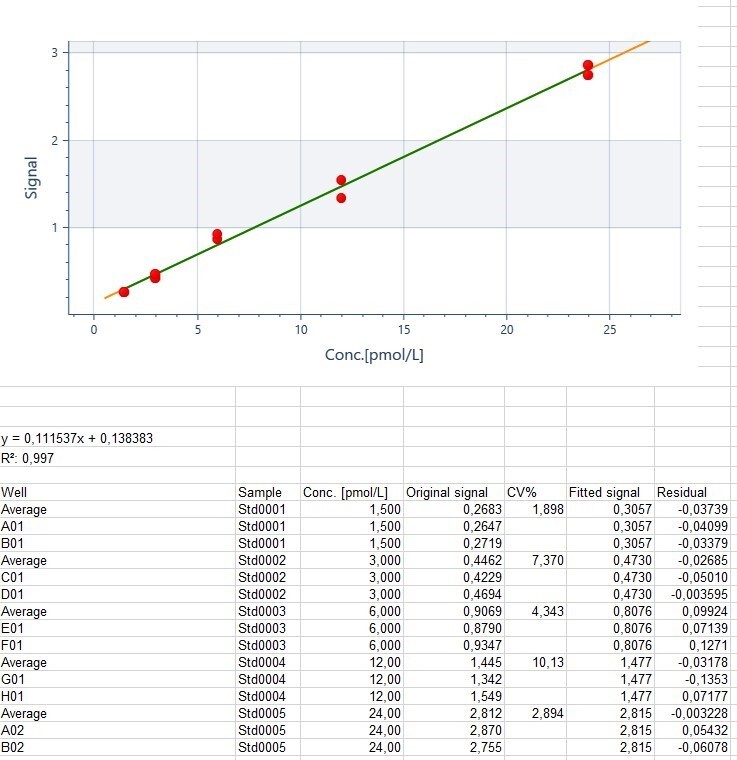

Supplement: Supplementary file 1 — Supplementary Material 1: Supplementary Figure S1. Standard curve of Cathepsin K ELISA. Representative standard curve obtained for Cathepsin K ELISA, demonstrating excellent linearity (R² = 0.997). Fitted regression line and residuals are shown. Signal values correspond to optical density at 450 nm (OD450). [file 12903_2025_7637_MOESM1_ESM.jpg]

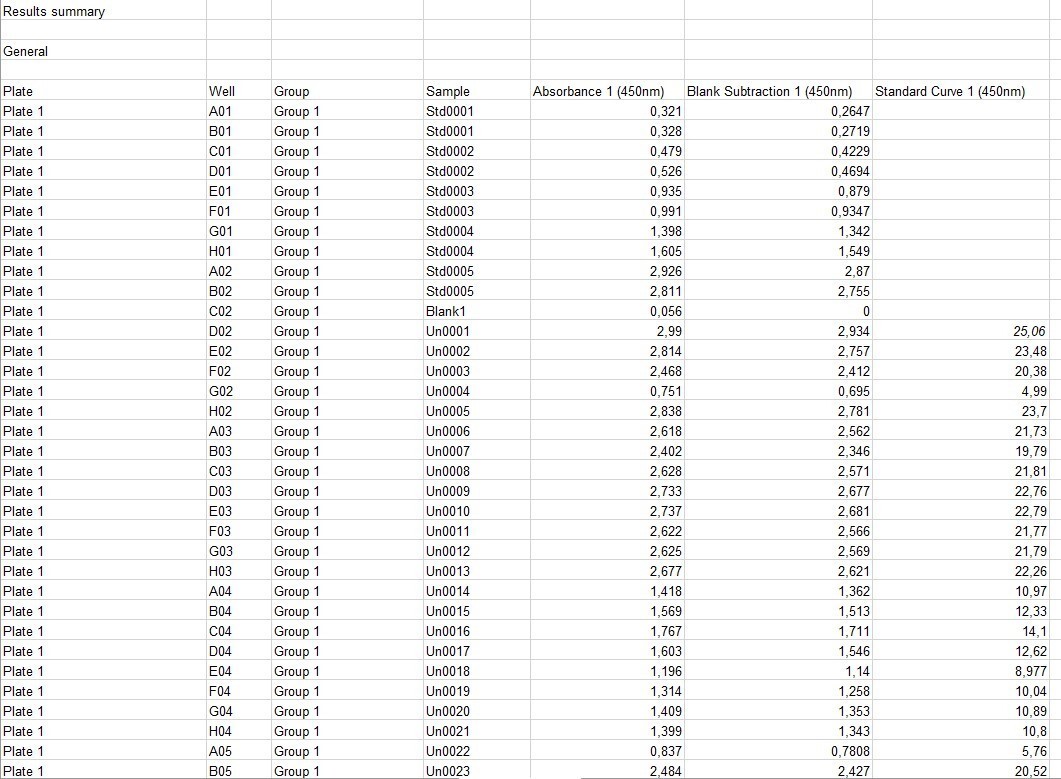

Supplement: Supplementary file 2 — Supplementary Material 2: Supplementary Figure S2. Representative absorbance values and standard curve-derived concentrations for Cathepsin K ELISA (Plate 1). Raw absorbance values at 450 nm, blank-subtracted signals, and corresponding concentrations interpolated from the standard curve are presented for both standard samples and representative unknown GCF samples. These data illustrate the transparency of the quantification process and confirm that all sample values fell within the dynamic range of the assay. [file 12903_2025_7637_MOESM2_ESM.jpg]
